# The Efficacy of Intra-articular Platelet-Rich Plasma Injection Versus Corticosteroid Injection in the Treatment of Knee Osteoarthritis: A Prospective Comparative Analysis

**DOI:** 10.7759/cureus.61040

**Published:** 2024-05-25

**Authors:** Sumbal Irshad, Usman Waleed, Muhammad Hassan Zafar, Muhammad Tayyab Ramzan, Muhammad Abdullah Tariq, Muhammad Hassan, Muhammad Ahmer Sohaib, Sana Liaquat, Sanwal Mehmood, Rana Shahzaib Ali, Tayyab Mumtaz Khan

**Affiliations:** 1 Internal Medicine, Philadelphia College of Osteopathic Medicine, Philadelphia, USA; 2 Orthopaedic Surgery, Benazir Bhutto Hospital, Rawalpindi, PAK; 3 Internal Medicine, Allama Iqbal Medical College, Lahore, PAK; 4 Rheumatology, Allama Iqbal Medical College, Lahore, PAK; 5 Orthopaedic Surgery, Sheikh Zayed Medical College and Hospital, Rahim Yar Khan, PAK; 6 Orthopaedic Surgery, Rawalpindi Medical University, Rawalpindi, PAK

**Keywords:** analysis, comparative, prospective, knee osteoarthritis, treatment, corticosteroids, platelet-rich plasma, intra-articular, efficacy

## Abstract

Background

Knee osteoarthritis (KOA) is the most typical cause of knee pain and impairment worldwide. It is typified by slow and progressive degeneration of the articular cartilage of the knee joint. Although KOA is being managed with a variety of therapies, the comparison of the effectiveness of different intra-articular injections in KOA treatment in Pakistan is still not thoroughly investigated. Therefore, the purpose of this current study is to compare the efficacy of intra-articular administration of platelet-rich plasma (PRP) and corticosteroids (CSs) in the treatment of KOA.

Methods

This prospective comparative study was performed among one hundred patients diagnosed with KOA in Benazir Bhutto Hospital, Rawalpindi, for one year from April 2022 to March 2023. Specified inclusion and exclusion criteria were employed for patient enrollment. Patients were divided into two equal groups through simple random sampling. Group A patients received an intra-articular injection of PRP solution whereas group B patients received an intra-articular injection of CSs. Informed consent and ethical approval were also acquired prior to data collection. A self-designed proforma based on interviews was used to collect data. The data analysis in Statistical Package for the Social Sciences (IBM SPSS Statistics for Windows, IBM Corp., Version 25.0, Armonk, NY) was carried out via descriptive statistics and an independent t-test.

Results

Women (N=71, 71%) had a higher prevalence of KOA than men (N=29, 29%). The means of study variables like age, Visual Analog Scale (VAS) score, and Western Ontario and McMaster Universities (WOMAC) score were 56.10 ± 8.70 years, 8.08 ± 1.6, and 70.08 ± 8.76 respectively. The frequency of KOA on the right side was 62% (N=62) while it was 38% (N=38) on the left side. In the study population, 69% (N=69) patients had grade II KOA, and 31% (N=31) patients had grade III KOA. At the first-month, second-month, and third-month follow-up visits, there were statistically significant differences in the mean scores of the WOMAC and VAS between the study groups. However, at the first-month follow-up visit, mean scores of VAS and WOMAC were lower in group B than in group A while these were lower in group A as compared to group B, at the second-month and third-month follow-up appointments.

Conclusions

Intra-articular infiltration of both PRP and CSs was efficacious in the treatment of KOA-related pain and functional limitations; however, overall improvement in the PRP group was higher than CS group.

## Introduction

Osteoarthritis (OA) is the most common disorder of the musculoskeletal system. It is distinguished by osteophyte production, articular cartilage degradation, and narrowing of joint space. OA symptoms include joint discomfort, edema, stiffness, restriction in joint movement, and nocturnal pain [1]. In the world, the prevalence of KOA is 9.6% in men and 18% in women [2]. According to a study, 28% of Pakistanis living in cities and 25% of those living in rural areas had KOA, indicating the significant frequency of the condition in this country [3]. Although OA can affect any joint; however, it typically affects the knees, hips, hands, and feet joints. Of all cases of OA, knee osteoarthritis (KOA) accounts for 23% of cases. KOA of the knees impacts up to 10% of adults after the age of 55 [4].

An imbalance in the homeostasis between the production and degeneration of the cartilaginous matrix is the processes that lead to KOA. One of the chemical alterations in KOA is chondrocyte senescence. The age-related decline in chondrocyte functions is referred to as chondrosenescence. Moreover, the proteolytic degradation of joint cartilage that results in KOA is further facilitated by different cytokines and growth factors [5]. Several variables, including advanced age, female sex, family history, obesity, weight lifting, knee trauma, inactive lifestyles, and deviant body postures, have been linked to the evolution of KOA [2,6].

According to estimates, 80% of KOA patients have mobility impairments and 25% of KOA patients are not even able to carry out their daily tasks [3]. The impacted population experiences reduced quality of social and financial facets of their lives as a result of difficulty in daily activities. Patients' mental health is also jeopardized by KOA, in addition to their physical condition. Patients with KOA are known to experience anxiety and depression frequently [7]. Additionally, it uses a lot of health-associated resources all over the world. The estimated annual cost of OA in the United States only is nearly 128 billion USD [8].

Treatment options for KOA include non-pharmacological measures (weight loss, diet changes, physical therapy, braces), systemic therapies (non-steroidal anti-inflammatory drugs (NSAIDs) and opioids), local therapies (topical analgesics and intra-articular injections), and knee replacement surgery as a last resort [1,4,5].

Intra-articular infiltration of platelet-rich plasma (PRP) activates platelets which inhibit the production of inflammatory mediators and enzymes while promoting the growth of chondrocytes, angiogenesis, cartilage shaping, and mesenchymal stem cells. On the other hand, corticosteroids (CSs) have comprehensive immunosuppressive and anti-inflammatory action. Intraarticular injections of CSs restrict T and B-cell activities and other inflammatory mechanisms, which reduce inflammatory changes in the joints and improve KOA symptoms [9-11].

Several international researches have documented the efficacy of intra-articular injections in the treatment of KOA; nevertheless, there are few studies that have compared the efficacy of PRP and CS intra-articular injections, particularly from Pakistan. Therefore, the purpose of this study was to compare the efficacy of intra-articular PRP and CS injection in the treatment of KOA.

## Materials and methods

Study design and study population

This comparative prospective study was conducted in the outdoor patient department (OPD) of orthopaedics in Benazir Bhutto Hospital, Rawalpindi, on one hundred patients diagnosed with unilateral KOA for one year from April 2022 to March 2023. Patients were divided into groups through simple random sampling (Lottery Method). Patients in group A were injected intra-articular injection of PRP whereas patients in group B were injected intra-articular injection of CSs.

Inclusion and exclusion criteria

Participants in the study were chosen based on a set of inclusion and exclusion criteria. Patients of both genders who had, age above 40 years, knee joint pain for at least six months, Kellgren Lawrence Ⅱ or Ⅲ grade of KOA, conservative treatment failure for the KOA within four months of its beginning, willingness to take part in the study, and who had attended three follow-up visits at first-month, second-month, and three-months after receiving intra-articular injections, were included in the study. Conversely, patients who had a history of advanced rheumatoid arthritis or any other knee deformities, previous knee surgeries, coagulopathies, intra-articular injections treatment during the previous six months in their affected knee joints, and who had shown reluctance for participation, were excluded from the study.

Ethical approval

The current study received ethical approval prior to its initiation from the Ethical Review Board (ERB) of Benazir Bhutto Hospital, Rawalpindi, Pakistan (ERB number: BBH.ERB.283/193). All participants gave their informed consent after elaboration about the objectives of the study.

Preparation and intra-articular administration of PRP and CSs

A total of 5ml of PRP solution was made by obtaining the 40ml of autologous blood from each participant. Around 2ml of ACD-A (anticoagulant citrate dextrose solution, solution A) was added to the blood sample for the prevention of clotting. Two centrifuge cycles each of six minutes were run at 1600 relative centrifugal force (RCF) and 2000 RCF respectively. The first was to separate erythrocytes and the second was to make a concentrate of platelets. For the activation of the PRP solution, 0.5ml of calcium gluconate (1g/10ml) was introduced in it. The prepared PRP was graded as P2xB12 as per DeLong's (PAW) classification. A 40mg of CSs (1 mL/40 mg of triamcinolone acetonide) was diluted in the 4ml 0.9% normal saline. Then after aspirating synovial fluid while the patient's knee was flexed to 90 degrees to confirm the synovial space, intra-articular injections of PRP solution (total 5ml) and CS (total 5ml) were administered through an anterolateral approach to the knee joint synovial space of the patients. Stringent aseptic procedures and the no-touch needle technique were used. All patients were recommended to wear kneecaps following injections.

Data collection

For data collection, a self-designed proforma was employed. It had two sections. The first section was about the age and gender whereas, in the second section, clinical evaluation of the patients was noted at baseline, first-month, second-month, and third-month follow-up visits. The Visual Analog Scale (VAS) and the Western Ontario and McMaster Universities (WOMAC) score were used to clinically evaluate the patients' pain and knee function in the OA-affected knee. The VAS uses a score range of 0 (no pain) to 10 (worst possible pain) to evaluate the intensity of pain. With scores ranging from 0 to 96, the three subscales of the WOMAC measure pain, stiffness in the joints, and function. Greater scores indicate more pain, more joint stiffness, and more limitation in joint function; lower values indicate less pain, stiffness in the joints, and limitation in joint function. International studies have also employed WOMAC and VAS [9,10]. In the second section of the proforma, information about the grade of KOA and the side of the affected knee was also recorded.

Sample size calculation and power of study

The superiority hypothesis was applied in the sample size calculation. Pain was measured using a VAS (VAS; range 0-10 points) at all follow-up visits. For the CS group, an average score of 8.20 with a standard deviation (SD) of 1.5 was assumed. This means that a total of 100 patients (50 in each group by keeping the 1:1 enrollment ratio) would need to be included in order to detect a reduction of 1.16 points in the PRP group in contrast to the CS group with a power of 80% and a two-sided significance level of 0.05. The probability of type 1 (Alpha) and type error 2 (Beta) were 5% and 20% respectively. Based on the results that have been published, a fixed difference of points was made in the VAS between the two groups' average scores and SD [12].

Data analysis

For data analysis, Statistical Package for the Social Sciences (IBM SPSS Statistics for Windows, IBM Corp., Version 25.0, Armonk, NY) was used. Both descriptive and inferential statistics were applied. For qualitative data, frequency and percentage were measured while for quantitative data, means with ± SD were calculated. To compare the means of the VAS and WOMAC values between two groups at various time intervals, the independent samples t-test was utilized. A p-value of less than 0.05 was considered statistically significant.

## Results

Table 1 shows the means of different variables in the research population.

**Table 1:** Means of demographic and clinical variables in the study population VAS: Visual Analog Scale; WOMAC: Western Ontario and McMaster Universities

Parameters	Mean	±Standard Deviation
Age in years	56.10	±8.70
VAS score	8.08	±1.60
WOMAC score	70.08	±8.76

Table 2 indicates that the frequency of KOA was higher among females and right-sided knee joints as compared to males and left-sided knee joints. Moreover, it also describes that the frequency of grade Ⅱ KOA was more than grade Ⅲ KOA in the study population.

**Table 2:** Frequency and percentage of knee osteoarthritis according to various study variables KOA: knee osteoarthritis

Parameters		Frequency and Percentage of KOA; N (%)
Gender	Female	71 (71%)
	Male	29 (29%)
Affected Side of Knee	Right	68 (68%)
	Left	32 (32%)
Grade of KOA	Grade Ⅱ	69 (69%)
	Grade Ⅲ	31 (31%)

Table 3 describes at the beginning of the study the variation in the means of VAS scores between group A and group B was insignificant. Conversely, the difference in the means of VAS scores between the research groups was significant at the first-month, second-month, and third-month follow-up appointments. It also shows that in group A, the VAS score mean was in descending course till the second-month follow-up appointment but at the third month of post-treatment, the VAS score began to increase gradually. On the contrary, in group B this declining trend of the VAS mean score was swift; however, it was of brief duration as the VAS score started to rise at the second-month follow-up visit.

**Table 3:** Means of VAS scores for study groups at various time intervals and an independent t-test analysis VAS: Visual Analog Scale

Parameters	VAS Scores		Independent t-test
Time Intervals	Group A	Group B	p-value
Baseline	8.00±1.50	8.16±1.08	1.02
First Month	3.90±1.10	2.60±1.23	0.02
Second Month	2.50±1.20	3.42±1.04	0.03
Third Month	2.70±1.02	3.70±1.76	0.04

Table 4 manifests at the baseline visit, the difference in the means of WOMAC scores between group A and group B was insignificant; nevertheless, the variation in the means of WOMAC scores at the first-month, second-month, and third-month post-intra-articular injections administration visits of the patients between the research groups was significant. It also explains that in group A, the WOMAC score mean was in a downward trend up to the second-month follow-up appointment; however, at the third-month follow-up visit, the mean of the WOMAC score initiated to rise slowly. On the contrary, in group B, although the declining course of the WOMAC mean score was faster, it was of short time duration as the WOMAC score mean commenced to increase in the second month of the post-treatment visit.

**Table 4:** Means of WOMAC scores for study groups at various time periods and an independent t-test analysis WOMAC: Western Ontario and McMaster Universities

Parameters	WOMAC Scores		Independent t-test
Time Intervals	Group A	Group B	p-value
Baseline	69.98±5.20	70.18±4.12	1.08
First Month	61.42±3.90	54.48 ±7.26	0.04
Second Month	50.50±5.67	56.38±8.21	0.03
Third Month	52.22±7.34	59.12±7.68	0.01

## Discussion

KOA management starts with non-pharmacological therapies such as exercise, patient counseling, physical therapy, and weight loss. After the failure of non-pharmacological remedies, oral analgesics (NSAIDs) are prescribed. As the disease worsens, other therapeutic approaches become important like intra-articular infiltration of CSs, hyaluronic acid, and PRP. However, there is still debate about the effectiveness of these intra-articular injections [1,10].

In the current study, the efficacy of intra-articular injections of PRP and CSs at various time points has been compared among the patients with KOA. Additionally, it has also manifested variations in KOA frequency according to gender, side of joint side, and KOA grade.

In the study population, KOA frequency was higher in females in contrast to males. Global researches have also revealed similar findings suggesting a higher prevalence of KOA among women than in men. Estrogen has anti-inflammatory action. After menopause, estrogen level goes down leading to an increased incidence of OA in women [2,6].

Regarding the variation in the distribution of KOA between the right and left sides of the body, it was more common on the right side of the knee joint than on the left. There are three possible causes for this asymmetry in KOA division: pathological, physiological, and constitutional. In addition, the dominant side of the body is often the right side in most individuals. As a result, there is a larger incidence of KOA on the right side of the knee joint due to its greater use and exposure to repetitive stress as compared to the left. This observation was endorsed by other investigations that had found more frequency of KOA on the right side [5,11].

Following the intra-articular infiltration of PRP and CSs into the synovial space of the patients' knee joints, the mean VAS scores were initially compared between the two groups at different time periods. According to the VAS scores at various points in time of group A, PRP had a long-term and progressive effect on KOA treatment. Whereas, according to the VAS scores at different patient visits of group B, CSs' action in the management of KOA was immediate; however, it was short-term. There was a statistically significant difference in the mean VAS scores at different time intervals between the research groups after intra-articular injection treatment.

WOMAC scores were also compared between research groups at different time points. The mean WOMAC scores for group A showed that PRP brings gradual and long-lasting betterment in KOA patients. Whereas, the mean WOMAC scores for group B demonstrated that these CSs give a quick but brief improvement in KOA patients. There was a statistically significant difference in the means of the WOMAC scores at various times between the study groups after intra-articular injection administration.

All over the world, many researches with findings comparable to current studies have been published in the literature. A study that was performed in Ireland suggested a similar role of PRP and CSs in the treatment of KOA [4]. A systemic review also suggested that PRP effects on KOA management last longer than CSs [9]. According to a study from Latvia, people with KOA get benefits slowly but in the long term from PRP. On the other hand, CSs bring rapid; however, short-term relief in patients with KOA [12]. Another study from Turkey supported these findings of the current study about the PRP and CSs' impact on KOA treatment [13]. Similarly, a study from the United Kingdom has also endorsed that PRP is superior to CSs in providing long-term benefits for KOA management [14]. A German study also reported similar findings about the higher impact of PRP on KOA [15].

CSs have instant but brief effects in the treatment of KOA because they reduce the inflammatory changes in joints for a short period of time. Whereas PRP aids in the repair of articular cartilage of joints by promoting the growth of chondrocytes, angiogenesis, cartilage shaping, and mesenchymal stem cells. This restoration process takes time and also lasts for a longer period of time which is why PRP brings betterment in KOA gradually; nevertheless, of prolonged duration [9,10]. As a result, we prescribe CSs for immediate care of KOA and PRP for long-term management of KOA.

This study has great importance as this is the only study that has compared the efficacy of PRP and CSs in KOA management in Rawalpindi, Pakistan. But it also has some limitations including its small sample size, follow-up visits at short time intervals, single intra-articular injection sessions of both PRP and CSs, and administration of intra-articular injections without ultrasound guidance. Therefore, the results of this study can only be extended to local populations and could have bias. In order to make these findings generalized and unbiased about the use of intra-articular injections of PRP and CSs in the treatment of KOA, researches with larger sample sizes, follow-up appointments at longer intervals, and multiple sessions of ultrasound-guided intra-articular injections are needed.

## Conclusions

This present study has shown that intra-articular injections of both PRP and CSs are sufficiently effective in the treatment of pain and functional limitations associated with KOA. CSs reduced both pain and functional limitations swiftly but for a lesser time period in contrast to PRP. However, PRP provided betterment in pain and functional restrictions slowly but for a longer duration in comparison to CSs. Moreover, it was also observed that there were statistically significant differences in the means of VAS and WOMAC between study groups at each of the three follow-up visits. In terms of the mean of VAS and WOMAC scores, it was found that during the first-month post-treatment visit, group B had lower scores than group A, while at the second-month and third-month follow-up appointments, group A had lower scores than group B. As a result, this study suggests PRP therapy plays a relatively better role in KOA management than CSs.
